# Extensive Circumferential Heterotopic Ossification Discovered at the Base of a Loop Ileostomy

**DOI:** 10.1155/2019/4036716

**Published:** 2019-12-02

**Authors:** Ahmad Bosaily, John Edminister, Samarchitha Magal, Mohammad Jamil, Amy Lynn, Glenn Hall

**Affiliations:** ^1^College of Medicine and Life Sciences, University of Toledo, Toledo, OH, USA; ^2^Department of Pathology, ProMedica Health System, Toledo, OH, USA; ^3^Department of Colorectal Surgery, ProMedica Health System, Toledo, OH, USA

## Abstract

Heterotopic ossification is a rare phenomenon defined by the formation of bone within nonossifying soft tissues. A rare variant of heterotopic ossification is heterotopic mesenteric ossification (HMO), in which there is involvement of the mesentery and surrounding intra-abdominal structures. There are only four previously reported cases of HMO involving an ileostomy. We present a case of HMO affecting an ileostomy which was discovered during elective stoma reversal in a 52-year-old male who required fecal diversion following perineal necrotizing fasciitis.

## 1. Introduction

Heterotopic ossification (HO) is a peculiar, benign disease characterized by organized bone formation in nonossifying muscle or soft tissue [1]. Ossification of soft tissue following orthopedic surgery, prolonged immobilization, or trauma has been well described. However, intra-abdominal cases of HO appear to be rare, with approximately 50 cases appearing in the medical literature [1]. Furthermore, there are even fewer reports of HO involving a stoma site [2–5]. In this report, we describe a case of HO involving a loop ileostomy.

## 2. Case

The patient is a 52-year-old African American male with an unwanted diverting loop ileostomy who was scheduled for an elective stoma reversal. Originally, the loop ileostomy was created for fecal diversion after a large perineal defect was made for the treatment of necrotizing fasciitis of the perineum. A high splenic flexure, foreshortened colonic mesentery, and morbid obesity precluded performing a diverting colostomy at that time. Of note, the patient had no family history of HO or abdominal surgical history prior to the ileostomy creation.

**Figure 1:**
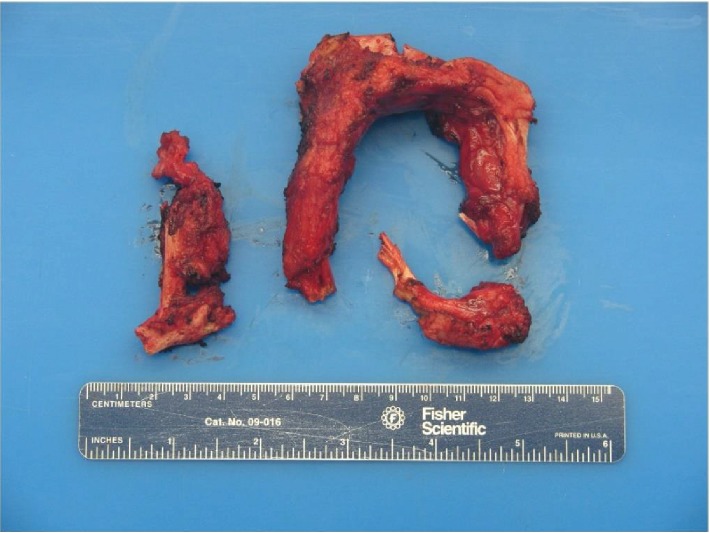
Gross specimen consists of three irregular portions of ragged skeletal muscle and bone measuring 5.5, 7, and 12.5 cm in the greatest dimension.

**Figure 2:**
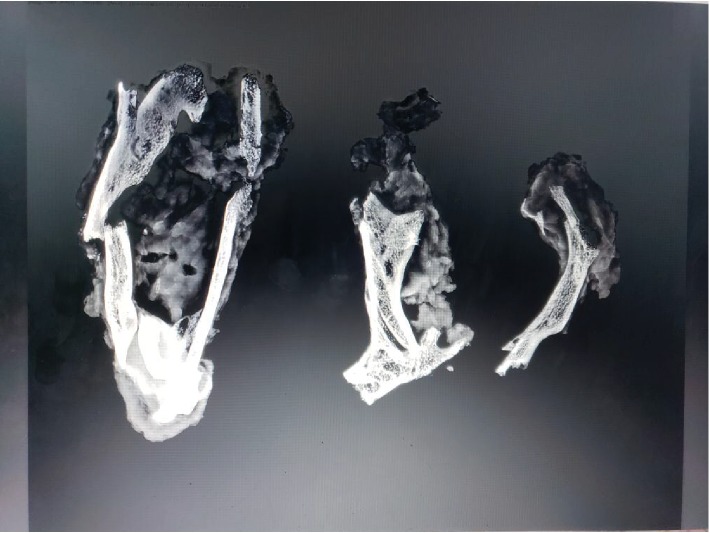
Specimen radiograph demonstrates both thick cortical and lacy trabecular bone irregularly distributed through the skeletal muscle fragments (12 cm, 7 cm, and 5.5 cm).

**Figure 3:**
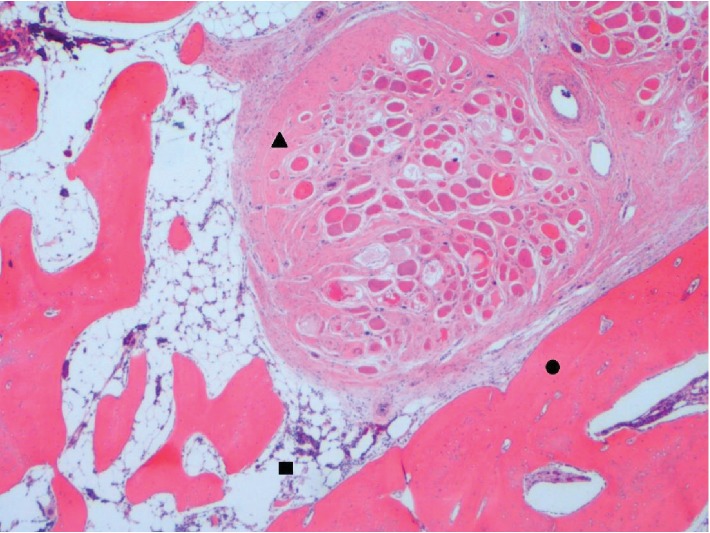
Trabecular bone (circle) with bone marrow (square) wrap around bundles of skeletal muscle (triangle) as is typical of myositis ossificans (2x magnification, H&E stain).

**Figure 4:**
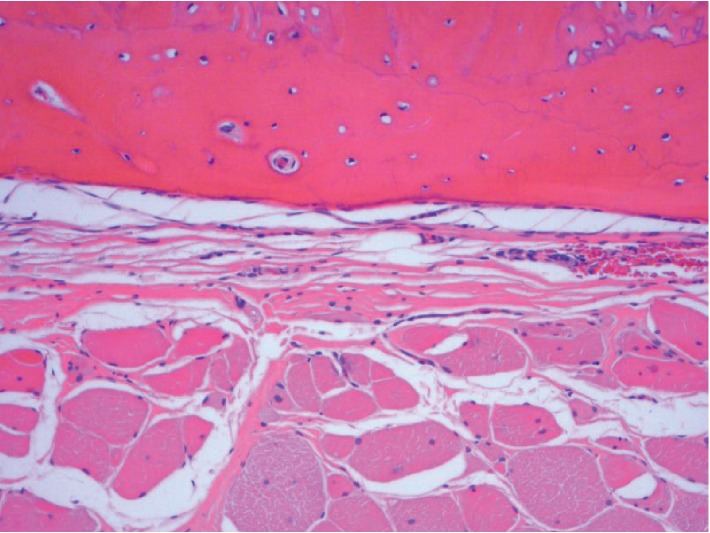
Cortical bone (top) demonstrating cement lines adjacent to skeletal muscle shown in cross section with peripherally located nuclei and myofibrils (10x magnification, H&E stain).

During the reversal of the loop ileostomy, a circumferential incision was made at the mucocutaneous junction. Dissection was carried towards the serosal surface of the small bowel with electrocautery. There were significant adhesions between the small bowel and surrounding soft tissue which was much denser than expected. At that time, the base of the ostomy was reached and a dense structure was identified by palpation. It was circumferential in both limbs of the loop ileostomy. It was hard and fixed with a bone-like consistency. We encountered difficulty in freeing the small bowel from this circumferential dense structure, and this, combined with the patient's body habitus, rendered it difficult to take the stoma down safely through the peristomal incision. As a result, we converted the operation to an open procedure using a lower midline incision to reverse the ileostomy. After significant lysis of adhesions, the ileostomy was reached from the midline incision, and the hard structure was excised circumferentially using a combination of sharp dissection with Metzenbaum scissors and Bovie electrocautery. Upon gross inspection, it was observed that the structure was seated at the level of the rectus abdominis muscle and formed a complete circle around the ileostomy. A portion appeared to have the consistency of thin connective tissue, while the majority of the specimen appeared osseous. The specimen grossly consisted of three irregular portions of ragged skeletal muscle and bone measuring 5.5, 7, and 12.5 cm in the greatest dimension (Figure 1). The skeletal muscle and bone were intertwined with flattened strands of bone interdigitating with the skeletal muscle fibers. Delicate, branching wisps of bone protruded from the specimen. No foreign body was identified. The specimen was radiographed using the Faxitron and showed both trabecular and cortical bone within the skeletal muscle (Figure 2). Microscopically, both thick cortical bone and trabecular bone with normocellular bone marrow were found within the skeletal muscle. Bone encircled and was immediately adjacent to skeletal muscle fibers (Figures 3 and 4).

The patient had follow-up visits after ileostomy reversal at weeks 2, 3, 7, 10, and 16. He developed transient fecal incontinence given the extensive nature of his perineal wound, which was successfully treated with pelvic floor biofeedback therapy by week 10. His ileostomy and midline wounds healed without abdominal pain, hernias, or signs of recurrence.

## 3. Discussion

Heterotopic ossification is a rare, benign disorder in which osteoblasts organize into discrete bone layers and deposit in nonskeletal tissues [1]. The phenomenon of HO was initially described in 1883 by Riedel in the context of a complication following spinal cord injury [6]. Previous literature reviews have described a 1901 case by Askanazy involving an abdominal scar [7]. The first reports of heterotopic ossification involving the intestinal mesentery were made by Lemershev et al. and Hansen et al. in 1983. In 1999, Wilson et al. coined the term heterotopic ossification to describe this pathology. Heterotopic ossification of soft tissue structures has been attributed to surgical, traumatic, and idiopathic etiologies involving different parts of the body [1, 3]. However, it appears that the amount of cases involving the intra-abdominal structures is low and that involvement of the stoma, as in our case, is even rarer [1, 4, 5, 8].

We describe a case of HO involving an ileostomy site, which we theorize could represent a rare subtype of intra-abdominal heterotopic mesenteric ossification (HMO). It appears that stoma involvement of HMO is a rare presentation of an already rare phenomenon, as only 4 other reports have specifically noted involvement of an ileostomy [2–5]. The morbidity associated with this case of HMO included a longer operation as necessitated by the need to convert to an open lower midline incision. Additionally, the patient suffered the less desirable cosmetic consequences of having this midline incision in addition to any potential risk of incisional hernia at the midline.

To date, there have been approximately 50 cases reported of HO [1, 9]. In 2012, Hirama et al. performed a literature review of 47 cases of HO in which they further classified the occurrence by location in the abdomen. Twenty-seven cases involved the mesentery, 8 cases involved the intestinal wall, and 6 cases involved the abdominal wall among other rarer sites [4]. Since that review in 2012, there have been a few more reports involving the mesentery. We also found recent cases involving relatively rare sites such as the abdominal wall and parietal peritoneum [1, 7, 9–13]. Considering all types of HMO, the mean age was 53.7 years old with at least 37 cases affecting males [14–16]. The lesions typically occurred after surgical interventions, but also sometimes without any previous surgeries, and when symptomatic, they most commonly presented as a small bowel obstruction [17].

Heterotopic ossification must be differentiated from other phenomena, such as dystrophic calcification and malignant bone tumors [1, 7]. Dystrophic calcification is a common pathology in which insoluble calcium salts are deposited in previously damaged tissue, despite a lack of osteoblastic activity [1]. Malignant tumors of the bone can be differentiated from HO by their growth pattern, both radiologically and histologically. The malignant bone tumors generally have indistinct borders on imaging, with histology showing central ossification and a lack of peripheral ossification [7]. This can be compared to the distinct HO lesions found on radiographs and primarily peripheral ossification seen on histology [7, 18].

There are three forms of HO: myositis ossificans progressiva (MOP), myositis ossificans circumscripta (MOC), and myositis ossificans traumatica (MOT). Myositis ossificans progressiva, also called fibrodysplasia ossificans progressiva or stone man disease, is an autosomal dominant connective tissue disorder caused by mutations in the *ACVR1/ALK2* gene, which codes for a bone morphogenetic protein type I receptor [19]. At birth, MOP is characterized by bilateral congenital deformities of the big toes and an otherwise normal appearance, with the heterotopic bone formation beginning only in childhood [20]. This disorder eventually progresses to immobilization due to extensive HO throughout the body and extra-articular joint fusion [19]. Myositis ossificans circumscripta is a disease in which there is isolated HO of a single muscle or muscle group; it is nonprogressive and can be idiopathic or occur following a trauma [21]. Myositis ossificans circumscripta and MOT are often used to define the same phenomenon, as they both can be products of trauma.

While MOT typically describes the phenomenon of HO in tissues adjacent to skeletal muscle, there have been reports of intra-abdominal MOT following abdominal surgery and/or trauma [22]. In these cases, an ossifying pseudotumor forms at the base of the mesentery and is often referred to as heterotopic mesenteric ossification, intra-abdominal myositis ossificans, mesenteritis ossificans, or heterotopic ossification of the intestinal mesentery [12]. Although the pathophysiology behind MOT remains unclear, there have been a few hypothesized theories. One theory speculates that MOT arises from an exaggerated inflammatory response that induces activation of mesenchymal osteogenic progenitor cells, thus producing HO in nonskeletal tissue [23]. Another theory proposes that disruption of the xiphoid process during abdominal surgery results in the seeding of these disturbed cells along the abdominal incision [24]. Risk factors associated with MOT are the same as those for severe inflammatory response: severe trauma to the extremities, higher disease burden, and traumatic brain injury [24].

Due to the rare nature of HMO and its nonspecific radiological appearance, it may be challenging to diagnose preoperatively without a biopsy. Imaging modalities may not be able to accurately distinguish between differential diagnoses such as contrast leakage or osseous neoplasia. This difficulty with radiologic identification is compounded by the only mild increase in soft tissue density that can be observed in early stages of heterotopic ossification [25]. Laboratory markers are nondiagnostic and often unrevealing, but some HO reports have described a nonspecific but sensitive elevation in alkaline phosphatase theorized to be due to increases in osteoblastic activity [26].

The exact natural history of heterotopic ossification regarding onset may depend on the location. The time from initial injury to development of ossification has been reported as early as 4 weeks following burn injury and as early as 3 months following elbow injury [27, 28]. One case report suggests that the time from initial injury could be as early as days for development of HO specifically affecting the mesentery [1]. The literature suggests that HO lesions carry no malignant potential; therefore, we presume that without intervention a lesion may remain benign indefinitely [1]. In our case, the time between ileostomy creation and reversal was 7 months.

The benign pathology of this condition lends credence to a conservative management if the lesions are asymptomatic, but a symptomatic case (i.e., bowel obstruction) may be an indication for surgical excision [1]. Excision of symptomatic lesions comes with the risks expected of operating on a patient [29]. It would seem intuitive to avoid unnecessary surgeries or incisions in a patient with a history of HO because surgery itself is a causative factor, and caution should be taken when operating on these patients due to reports of recurrence or further progression [1, 9]. Prophylactic use of nonsteroidal anti-inflammatory drugs, bisphosphonates, or radiation therapy in high-risk patients have been shown to reduce the incidence of HO [30–32].

Heterotopic ossification must be distinguished from other pathologies that may present similarly. As in our case, the surgical team may initially confuse HO with a foreign body due to the shape and location of the ossification. This is an important consideration because a surgeon may choose to simply leave ossification behind or at most perform an incisional biopsy, as opposed to removal if it is a foreign body. Furthermore, a tissue capsule usually forms around a foreign body making its removal more straightforward. Knowledge of such cases may deter the surgeon from performing further dissection due to a benign pathology such as HMO [1]. However, in our case, dense adhesions between the ossification and small bowel required extensive dissection and removal of the entire osseous structure to allow for ileostomy reversal. An on-table X-ray may also be used to help differentiate between a foreign body and ossification.

## 4. Conclusion

We report a rare case of HMO that was identified at loop ileostomy takedown. A thorough literature review revealed only four previous cases involving an ileostomy. Heterotopic mesenteric ossification can result in serious complications, including but not limited to small bowel obstruction, fistula, sepsis, and death [33]. However, surgical excision appears to be an acceptable and suitable treatment in symptomatic patients. This is a particularly rare condition described in the literature, though it may be underreported. As such, further investigation of the etiology, diagnosis, prevention, and treatment of HMO is essential to delineate appropriate prophylactic and therapeutic options for these patients.
